# TRIM47 is up-regulated in colorectal cancer, promoting ubiquitination and degradation of SMAD4

**DOI:** 10.1186/s13046-019-1143-x

**Published:** 2019-04-12

**Authors:** Qian Liang, Chaotao Tang, Mingyu Tang, Qingwei Zhang, Yunjie Gao, Zhizheng Ge

**Affiliations:** Division of Gastroenterology and Hepatology, Key Laboratory of Gastroenterology and Hepatology, Ministry of Health, Renji Hospital, School of Medicine, Shanghai Jiao Tong University, Shanghai Institute of Digestive Disease, Shanghai, 200001 China

**Keywords:** Colorectal carcinoma, E3 ubiquitin ligase, Proliferation, Metastasis, TRIM47

## Abstract

**Background:**

Tripartite motif 47 (TRIM47), a member of the TRIM family proteins, plays a key role in many types of cancers including colorectal cancer (CRC). We found that levels of TRIM47 mRNA and protein were increased significantly in colorectal tumors compared with nontumor tissues and the increased levels were associated with advanced tumor stage and poor outcome.

**Methods:**

We used quantitative polymerase chain reaction and western blot to measure levels of TRIM47 mRNA and protein in human colorectal cancer and paired normal tissues. TRIM47 was knocked down and overexpressed in colorectal cancer cells, and the effects on cell proliferation, migration and growth of xenograft tumors in nude mice were assessed. The signaling pathways were examined by western blot and immunoprecipitation assays.

**Results:**

TRIM47 promoted CRC proliferation and metastasis in vitro and in vivo as an oncogene. Mechanistically, TRIM47 interacted physically with SMAD4, increasing its ubiquitination and degradation. Loss of SMAD4 leaded to up-regulation of CCL15 expression and caused growth and invasion in human CRC cells through the CCL15-CCR1 signaling. Moreover, TRIM47 overexpression played a role in CRC chemoresistance in response to 5-FU therapy.

**Conclusions:**

Our study demonstrated a functional role of the TRIM47-SMAD4-CCL15 axis in CRC progression and suggested a potential target for CRC therapy.

**Electronic supplementary material:**

The online version of this article (10.1186/s13046-019-1143-x) contains supplementary material, which is available to authorized users.

## Introduction

Over 1.8 million new colorectal cancer(CRC) cases and 881,000 deaths are estimated to occur in 2018, accounting for about 1 in 10 cancer cases and deaths. Overall, colorectal cancer ranks third in terms of incidence but second in terms of mortality [1]. Incidence and mortality rates have been declining for several decades in the United States because of historical changes in risk factors, the introduction and dissemination of screening tests, and improvements in treatment [2]. However, colorectal cancer incidence rates are about 3-fold higher in transitioned versus transitioning countries [1]. Assessing incidence and mortality trends, Arnold et al [3] identified 3 distinct global temporal patterns linked to development levels among which China has increases in both incidence and mortality in the most recent decade. The increase in mortality is predicted to continue until at least 2035, with a widening gap between lower and higher resource regions [4]. Increasing our understanding of CRC pathogenesis could lead to new strategies for diagnosis and treatment to reduce the global burden of CRC.

Emerging clinical evidence shows that the disorder of ubiquitin-mediated degradation of oncogene products or tumour suppressors is likely to be involved in the aetiology of carcinomas [5]. Of the ubiquitin–proteasome system(UPS) components, the E3 ubiquitin ligase, which can recognize substrates with the most specific, has been regarded as the potential diagnosis and therapeutic target in cancer [6]. Members of the family of tripartite motif (TRIM)-containing proteins could be defined as E3 ubiquitin ligases as they contain a RING-finger domain, although not all proteins containing a RING-finger domain function as E3 ubiquitin ligases [7]. Recent studies have indicated that changes in expression levels could disrupt the balance of TRIM proteins in the cell and alter ubiquitination of various proteins to produce irregular cellular signaling that could lead to tumorigenesis [8]. For example, TRIM proteins including TRIM59 [9], TRIM37 [10], TRIM25 [11], TRIM65 [12] and TRIM27 [13] were shown to be involved in gastric, breast, prostate, bladder urothelial and colorectal cancer through the ubiquitin-mediated degradation, indicating crucial roles of the TRIM family in tumorigenesis. TRIM47, defined as E3 ubiquitin ligases, was first identified as over-expressed in astrocytomas. TRIM47 was localized to 17q24–25, a region that is frequently gained or amplified in a number of other tumor types [14]. Higher TRIM47 expression was reported to be associated with human prostate cancer [15] and non-small cell lung cancer [16]. However, the pathologic and clinical role of TRIM47 has not been revealed in colorectal cancer.

In this study, we first reported that TRIM47 overexpression is common and related with poor prognosis of CRC patients (Additional file 2). Functionally, TRIM47 facilitates the proliferation and metastasis of colorectal cancer cell in vitro and in vivo. Mechanistically, TRIM47 promotes the ubiquitination and degration of SMAD4 by interacting with SMAD4, and further increases the levels of C-C motif chemokine ligand 15 (CCL15) and C-C motif chemokine receptor 1 (CCR1), ultimately causing poor outcome of colorectal cancer.

## Materials and methods

### Clinical specimens

Clinical colorectal cancer samples were obtained from Renji Hospital affiliated to Shanghai Jiaotong University School of Medicine (Shanghai, China) between 2012 and 2017, cohort 1 with 100 fresh tissues, cohort 2 with 180 formalin-fixedparaffin-embedded tissues, respectively. No patient received preoperative treatment, and all provided informed consent. Patients were pathologically and clinically diagnosed as colorectal cancer. This study was approved by the ethics committee of Shanghai Jiaotong University School of Medicine, Renji Hospital.

### Cell culture and treatment

Human colorectal cancer cell lines HCT116, HT29, SW480, RKO, SW620, Caco2, LoVo and SW1116 (ATCC, USA) were cultured in RPMI-1640 medium (Gibco, Carlsbad, CA) supplemented with 10% fetal bovine serum (FBS) and 1% penicillin-streptomycin sulfate (Gibco, Carlsbad, CA) at 37 °C in a humidified 5% CO_2_ atmosphere. Human normal colorectal epithelial cell FHC was cultured in DMEM/F12 medium (Gibco, Carlsbad, CA) supplemented with 25 mM HEPES, 10 ng/ml cholera toxin, 0.005 mg/ml insulin, 0.005 mg/ml transferrin, 100 ng/ml hydrocortisone and 10% FBS at 37 °C in a humidified 5% CO_2_ atmosphere.

Colorectal cancer cells were transfected with two siRNAs targeting human TRIM47 gene and a nonspecific siRNA as negative control, using the DharmaFECT1 Transfection Reagent (GE Healthcare, UK, T-2001-03). The TRIM47-overexpressing plasmid and the empty plasmid acting as negative control were transfected into cells using the FuGENE Transfection Reagent (Promega, USA, E2311). The siRNAs were purchased from GenePharma Technologies (Shanghai, China) and the plasmids from GENEray Biotechnology (Shanghai, China). The plasmid vector was pCDNA3.1(+) and the cloning site was HindIII/EcoRI. The expression plasmids encoding TRIM47 with deletion of the RING domain (△RING) was constructed by truncating 1–84 amino acid [17]. TRIM47 overexpression plasmids and the △RING domain plasmids used in the ubiquitination assay were tagged with Flag. The sequences of the siRNAs were as follows: #1: sense, 5′-CCAGGGACUAUUUCCUCAATT-3′ and antisense, 5′-UUGAGGAAAUAGUCCCUGGTT-3′; #2: sense, 5′- GCAGCUGUUUGGAACCAAATT-3′ and antisense, 5′- UUUGGUUCCAAACAGCUGCTT -3′.

### Total RNA extraction and real time RT-PCR

Total RNA was extracted from patient tissues (cohort 1) and cells using Trizol Reagent (Takara, Japan, 9108) in a ventilator and according to manufacture’s guideline. RNA was reverse-transcribed into cDNA using the PrimeScript RT Reagent Kit (Takara, Japan, DRR037A). Quantitative real-time PCR was performed using SYBR Premix Ex Taq™ II (Takara, Japan, DRR820A) in an ABI StepOnePlus system (Applied Biosystems Inc., USA). The primers were provided by Sheng gong Company, Shanghai. The sequences of primers were as follows: TRIM47, 5′-GCTTCAGGAGGCTGAGCAGT-3′ and 5′-TCTGCTACGGCTGCACTCTT-3′; SMAD4, 5′-ACAAGTAATGATGCCTGTCTGA-3′ and 5′-CTCCCATCCAATGTTCTCTGTA-3′; GAPDH, 5′-GCATTGCCCTCAACGACCAC-3′ and 5′- CCACCACCCTGTTGCTGTAG-3′. The expression of target genes was normalized by GAPDH acting as an internal control. The comparative CT (ΔΔCT) method was used to analyze fold change.

### Cell proliferation and transwell assay

Cell proliferation was estimated by the Cell Counting Kit-8 (CCK8, Dojindo Molecular Technologies, Japan, 3000 T). Control and treated cells were seeded onto 96-well plates at the initial density of 3000 cells/well. Then, CCK8 reagent (10 μl/well) was added to each well at the time points of 24 h, 48 h, 72 h and 96 h. After 2 h incubation at 37 °C, we use a microplate reader (Thermo Scientific, USA) to obtain the cells absorbance at 450 nm.

For colony formation assay, cells were plated in a 6-well plate at a density of 700 per well. The cells were continuously cultured for approximately 2 weeks after transfection until evident colony formation was observed. Colonies were fixed with 4% paraformaldehyde (Dingguo Biotechnologies, China, AR-0211) and stained by 0.1% crystal violet (BBI Life Sciences Corporation, China, CB0331).

To perform transwell assay, we precoated the transwell chambers (8-μm pore size; Millipore, USA, MCEP24H48) with Matrigel (BD Biosciences, USA, 354234) that diluted in 1:4 proportion with serum-free medium. 2.0 × 10^5^ cells suspended in serum-free medium were plated on the top of each chamber, while medium containing 20% FBS was put in the lower chamber as a chemoattractant. After 48 h incubation, cells that did not pass through the filter were removed by a cotton swab, and cells that pass through the filter were fixed by 4% paraformaldehyde (Dingguo Biotechnologies, China, AR-0211) and stained by 0.1% crystal violet (BBI Life Sciences Corporation, China, CB0331). The migrated cells could be counted using a microscope.

### Western blot

Cells were lysed with RIPA lysis buffer (Beyotime, China, P0013C) in the presence of a protease inhibitor mixture (protease inhibitor, phosphatase inhibitor, PMSF; KangChen, Shanghai, China, KC-440) on ice for 30 min. Protein concentration was determined using a BCA protein assay kit (Bio-Rad, Hercules, USA, 23227). For western blotting, 60 μg of proteins were separated on 10% SDS-polyacrylamide gels and transferred to PVDF membranes (Millipore, Bedford, USA, ISEQ00010). The membranes were then blocked in 5% nonfat milk at room temperature for 1 h and incubated with the primary antibodies at 4 °C overnight. The horseradish peroxidase-conjugated secondary antibodies (1:5000; KangChen, Shanghai, China, KC-RB-035) were used to incubate the membranes. GAPDH was used as a protein loading control. Finally, the ECL detection system (SuperSignal West Femto Maximum Sensitivity Substrate, Thermo Fisher Scientific, USA, 34096) was used for visualization. Sources of antibodies and the concentration used were as follows: rabbit anti-TRIM47 (1:1000, Sigma, USA, SAB2108331), rabbit anti-SMAD4 (1:1000, CST, USA, 46535), goat anti-CCL15 (1:2000, R&D Systems, USA, 628-LK-025), goat anti-CCR1 (1:1000, Abcam, USA, ab139399), mouse anti-Ubiquitin (1:1000, Active Motif, USA, 39741), rabbit anti-Cyclin D1 (1:1000, CST, USA, 2978), rabbit anti-c-Myc (1:1000, CST, USA, 13987), rabbit anti-Snail (1:1000, CST, USA, 3879), rabbit anti- MMP9 (1:1000, CST, USA, 13667), rabbit anti-CDH1 (1:1000, CST, USA, 3195), mouse anti-Flag (1:1000, Abcam, USA, ab18230), GAPDH (1:3000; KangChen, Shanghai, China, KC-5G5).

### Immunohistochemistry

The tissue microarray sections were rehydrated and treated with hydrogen peroxide for 15 min at room temperature to suppress endogenous peroxidases, followed by antigen retrieval. After being blocked with goat serum for 30 min, the tissue sections were incubated with primary antibodies at 4 °C overnight, followed by incubation with a peroxidase-labeled secondary antibody for 30 min at room temperature. After the diaminobenzidine (DAB; Maixin Biotech, China, DAB-0031) reaction was developed, the slides were the counterstained with hematoxylin. Colorectal cancer and normal tissue sections were stained with primary antibodies against TRIM47 (Abcam, USA, ab155549), SMAD4 (CST, USA, 46535), Ki67 (CST, USA, 9449), Elivision plus Polyer HRP (Mouse/Rabbit) IHC Kit (Maixin Biotech, China, KIT-9902).

The tissue slides were inspected by two investigators independently. Protein expression was assessed according to the intensity and extent of staining (the grade of intensity was measured on a scale of 0–3: 0, no staining; 1, weak staining; 2, moderate staining; 3, strong staining; the percentage of positive cells was measured on a scale of 0–4: 0, none; 1, 1–25%; 2, 26–50%; 3, 51–75%; 4, > 75%). The final staining score was given by multiplying the extent and grades of intensity staining. Therefore, the scores were ranked from 0 to 12. Ultimately, the protein expression was sorted into high expression and low expression by the median of the sample size.

### Co-immunoprecipitation (co-IP) and ubiquitination assay

Four micrograms normal rabbit IgG (Beyotime, China, A7016) or IP antibody, 40 μl suspended IP Matrix (Santa Cruz, USA, sc45039) and 500 μl PBS were incubated at 4 °C on a rotator for at least an hour. The mixture was then centrifuged and washed three times with PBS in the presence of a protease inhibitor mixture (protease inhibitor, phosphatase inhibitor, PMSF; KangChen, Shanghai, China) and then discard supernatant carefully. Cells that transfected for 48 h were lysed and transferred to the matrix, incubating at 4 °C on a rotator overnight. Next, the matrix was centrifuged and washed for five times. SDS-PAGE sample loading buffer (Beyotime, China, P0015) was added to the immunoprecipitates followed by boiling for 10 min at 100 °C. The IP and input proteins were detected by western blot.

### Immunofluorescence

Pretreated HCT116 cells were plated in eight-well chamber slides (Nunc, Denmark) 24 h prior to experiment. The cells were probed with corresponding antibodies for 1 h at room temperature and incubated with Alexa-488-conjugated donkey anti-mouse IgG (Invitrogen, USA, A-21202), followed by Alexa-594-conjugated donkey anti-rabbit IgG (Invitrogen, USA, R37119). The slides were mounted in DAPI Fluoromount-G (SouthernBiotech, Birmingham, USA, 0100–20). The images were captured using a laser-scanning confocal microscope (LSM-710, Zeiss, Germany).

### In vivo experiments

To illuminate the effect of TRIM47 on tumor growth in vivo, 4-week-old male BALB/c nude mice were purchased from Slac Laboratory Animal (Shanghai, China). In order to generate subcutaneous tumor, HCT116 cells (1.0 × 10^7^) were injected subcutaneously into the right oxter of all mice to establish the colorectal cancer xenograft model. A week later, mice were divided into three groups randomly, injecting PBS, control shRNA adenovirus or TRIM47 shRNA virus respectively by the way of multipoint intratumoral injection every 2 days for 2 weeks. The length and width of tumors were measured with a caliper and tumor volume (mm^3^) was calculated with the formula: tumor volume (mm^3^) = longer diameter×shorter diameter^2^/2. Then mice were sacrificed and the tumor tissues were isolated and frozen in liquid nitrogen or fixed in formalin immediately.

To investigate the effect of TRIM47 on tumor metastasis in vivo, we established a colorectal cancer metastasis model by injecting HCT116 cells (2.0 × 10^7^) subcutaneously into the right flank of 4-week-old male BALB/c nude mice. After a week, mice were divided into three groups randomly and injected with PBS, control shRNA and TRIM47 shRNA virus respectively twice a week for 14 weeks. To examine lung metastasis, the lung tissues were isolated from the nude mice for HE staining.

To explore the role of TRIM47 in chemoresistance in vivo, we used SW480 cells (1.0 × 10^7^) in the xenograft experiments. Mice were divided into four groups randomly and received intratumoral injection with PBS, TRIM47-overexpressing adenovirus, 5-Fu (50 mg/kg), TRIM47 -overexpressing adenovirus & 5-Fu every 3 days for 3 weeks. After 3 weeks, all mice were sacrificed and the subcutaneous tumors were collected and weighted. All study procedures were approved by the Institutional Animal Care and Use Committee of Renji Hospital, School of Medicine, Shanghai Jiaotong University.

### Statistical analysis

The statistical analysis was carried out with the SPSS 21.0 software (SPSS Inc., Chicago, USA). The paired-sample*t* test or independent-sample*t* test was performed to compare the difference between the measurement of two groups. The Chi-square test or Fisher’s exact test was used to determine the strength of correlations among TRIM47 expression and the clinicopathological data. The overall survival curve was constructed by using the Kaplan-Meier method and analyzed by the log-rank test. Cox proportional hazards regression model was used to identify the prognostic factors by univariable and multivariable analysis. All data were from at least three independent experiments and were showed as the means ± standard error (SE) that was presented with error bars in the scatter plots, line charts and bar graphs. A two-sided*P*-value under 0.05 was considered statistically significant in all tests.

## Results

### TRIM47 is overexpressed in colorectal cancer and correlated with disease progression as well as poor prognosis

**Fig. 1:**
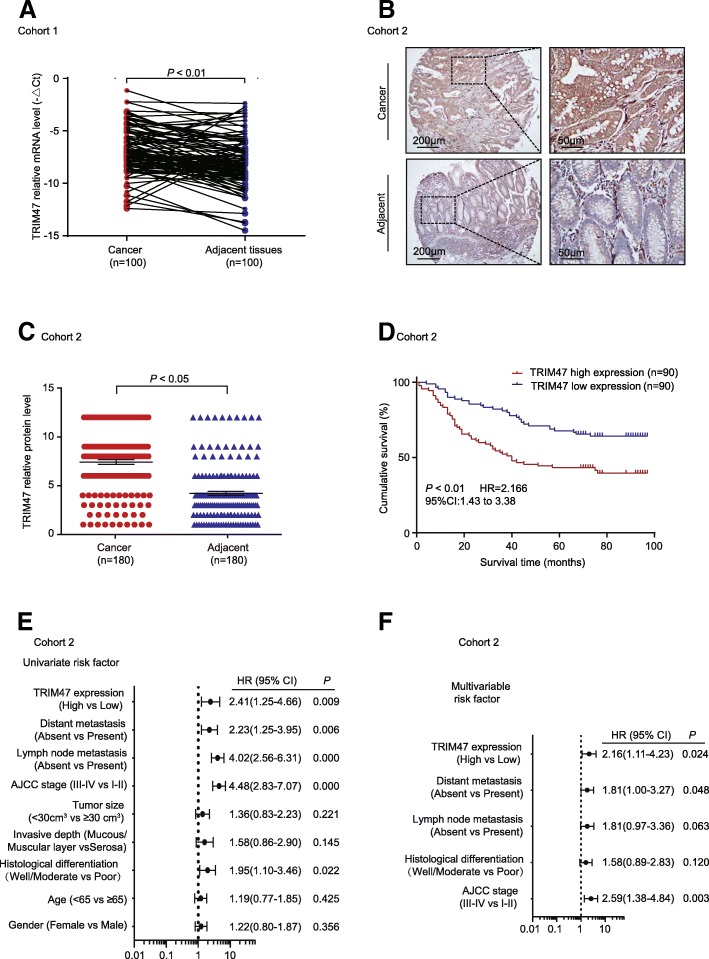
TRIM47 is overexpressed in colorectal cancer and correlated with disease progression as well as poor prognosis. **a** TRIM47 mRNA expression in colorectal cancer and the paired adjacent normal tissues of Renji cohort 1 (*n* = 100, non-parametric Mann-Whitney test, *P* < 0.01). **b** Representative images of TRIM47 protein expression in colorectal cancer tissues and paired adjacent tissues using IHC in Renji dataset (Cohort 2, *n* = 180). **c** Statistical analysis of TRIM47 protein expression in colorectal cancer tissues and paired adjacent tissues using IHC staining in Renji dataset (Cohort 2, *n* = 180, nonparametric Mann–Whitney test, *P* < 0.05). **d** Survival analysis was conducted between patients with high and low expression levels of TRIM47 in Renji cohort 2 (*n* = 180, Log-rank test, *P* < 0.01). **e** Univariate regression analysis in Cohort 2 (All the bars correspond to 95% confidence intervals). **f** Multivariable regression analysis in Cohort 2 (All the bars correspond to 95% confidence intervals)

To determine the significance of TRIM47 in colorectal cancer, the expression level of TRIM47 was examined using qRT-PCR in 100 cases of CRC patients (Additional file 3) of Renji hospital (Cohort 1). Real time PCR showed that TRIM47 expression was remarkably up-regulated in 76 of the 100 (76%) colorectal cancer samples compared with the paired adjacent normal tissues (*P* < 0.01, Fig. 1a). In order to verify the clinical significance of TRIM47, we analyzed the relationship between TRIM47 expression and clinicopathological features of CRC patients in Cohort 1. As summarized in Table 1, TRIM47 mRNA level was notably associated with tumor size (*P* = 0.019), histological differentiation (*P* = 0.013), T classification (*P* = 0.023), TNM stage (*P* = 0.041) and lymph node metastasis (*P* = 0.041); whereas no significant relevance was found with age, gender, tumor location, invasive depth, distant metastasis and vascular invasion. In addition, TRIM47 protein expression was measured by immunohistochemical staining assay in 180 paraffin-embeddded colorectal cancer and adjacent normal tissues (Cohort 2). TRIM47 protein expression was higher in colorectal cancer tissues than the adjacent tissues (*P* < 0.05, Fig. 1b and c). Next, we explored whether the higher expression of TRIM47 in colorectal cancer was related with poor prognosis. The Kaplan-Meier analysis demonstrated that TRIM47 expression was dramatically associated with shortened CRC patients’ overall survival in Renji cohort 2(*P* < 0.01, Fig. 1d). Moreover, univariate and multivariate regression analysis of cohort 2 were performed to identify the risk factors associated with patient overall survival. The results revealed that TRIM47 expression was an independent predictor of colorectal cancer aggressiveness with significant hazard ratios for predicting clinical outcome (Fig. 1e and f). These data indicated that TRIM47 overexpression may be a predictor for diagnosis and prognosis in CRC patients and herefore we chose to focus our experimental research on TRIM47.

**Table 1:** Correlations between expression of TRIM47 protein and clinicopathological factors

Clinicopathological feature	Total 100	Expression of TRIM47		*P* value (χ^2^ test)
		Low (*n* = 24,24%)	High (*n* = 76,76%)	
Age(years)				
< 65	48	10(41.7)	38(50.0)	0.476
≥65	52	14(58.3)	38(50.0)	
Gender				
Male	67	18(75.0)	49(64.5)	0.339
Female	33	6(25.0)	27(35.5)	
Tumor location				
Rectum	47	11(45.8)	36(47.4)	0.895
Colon	53	13(54.2)	40(52.6)	
Tumor size				
<30cm^3^	68	21(87.5)	47(61.8)	0.019
≥30 cm^3^	32	3(12.5)	29(38.2)	
Histological differentiation				
Well/moderate	23	10(41.7)	13(17.1)	0.013
Poor	77	14(58.3)	63(82.9)	
T classification				
T1,2	21	9(37.5)	12(15.8)	0.023
T3,4	79	15(62.5)	64(84.2)	
TNM stage(AJCC)				
Stage I, II	43	6(25.0)	37(48.7)	0.041
Stage III, IV	57	18(75.0)	39(51.3)	
Invasive depth				
Mucous/muscular layer	20	8(33.3)	12(15.8)	0.080
Serosa	80	16(66.7)	64(84.2)	
Lymph node metastasis				
Absent	57	18(75.0)	39(51.3)	0.041
Present	43	6(25.0)	37(48.7)	
Distant metastasis				
Absent	86	22(91.7)	64(84.2)	0.508
Present	14	2(8.3)	12(15.8)	
Vascular invasion				
Absent	69	19(79.2)	50(65.8)	0.217
Present	31	5(20.8)	26(34.2)	

### TRIM47 promotes the proliferation and metastasis of colorectal cancer cell in vitro and in vivo

**Fig. 2:**
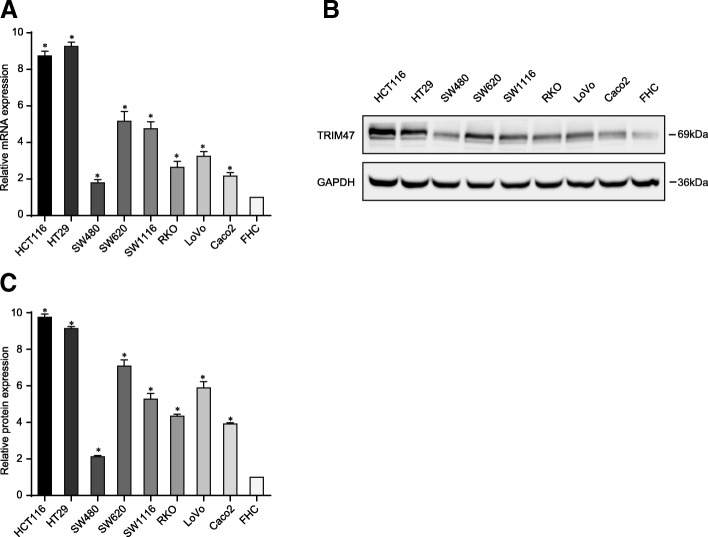
The expression of TRIM47 protein in human colorectal cancer cells. **a** Real time PCR was performed in 8 different colorectal cancer cells and normal colorectal cell FHC (*n* = 3). **b** and **c** Western blot was performed in 8 different colorectal cancer cells and normal colorectal cell FHC (*n* = 3)

To clarify the biological functions of TRIM47, functional assays were performed. First, we detected the expression of TRIM47 in 8 different colorectal cancer cells and normal colorectal cell FHC by real time PCR and western blotting. The results showed HCT116 and HT29 expressed higher levels of TRIM47, while SW480 expressed lower levels of TRIM47 (Fig. 2a-c). Therefore, we transfected HCT116 and HT29 with TRIM47 siRNAs and SW480 with TRIM47-overexpressing plasmid, respectively.

**Fig. 3:**
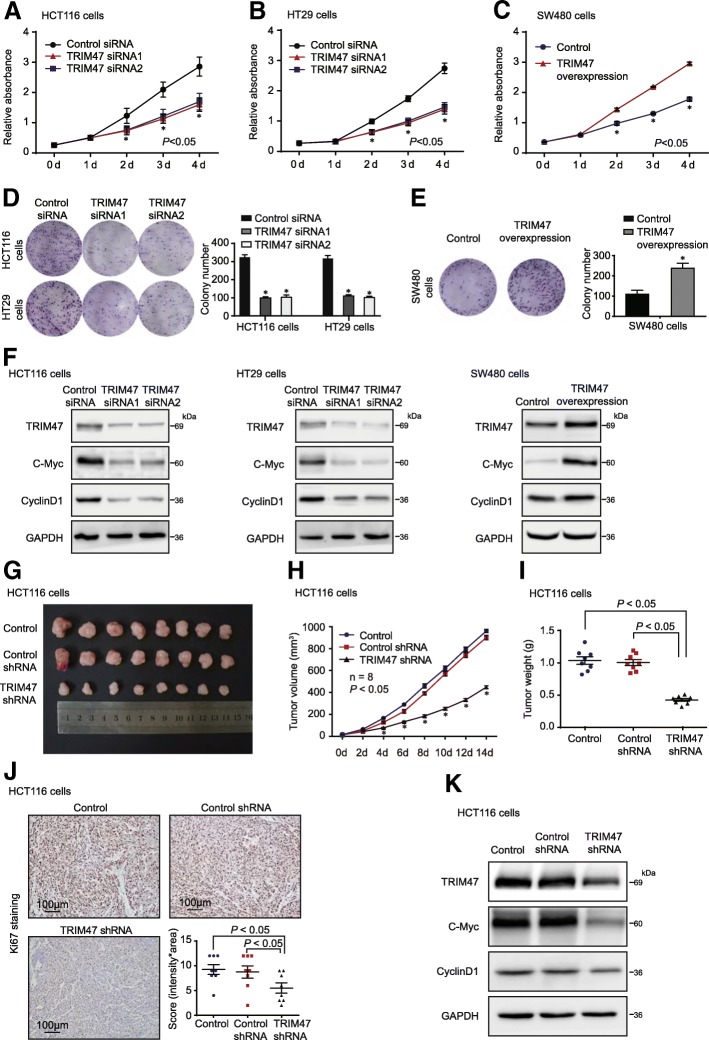
TRIM47 promotes proliferation of colorectal cancer cells in vitro and in vivo. **a** and **b** Cell proliferation assay was performed in HCT116 and HT29 cells transfected with control siRNA and TRIM47 siRNAs (*n* = 3, non-parametric Mann-Whitney test, *P* < 0.05). **c** Cell proliferation assay was performed in SW480 cells transfected with vector plasmids and TRIM47 overexpression plasmids (*n* = 3, non-parametric Mann-Whitney test, *P* < 0.05). **d** and **e** Colony forming assay was performed in HCT116, HT29 and SW480 cells (*n* = 3, non-parametric Mann-Whitney test, *P* < 0.05). **f** Proliferation related proteins c-Myc and Cyclin D1 were detected in HCT116, HT29 and SW480 cells by western blot (*n* = 3). **g** Representative images of tumors in nude mice bearing colorectal cancer cells treated with PBS, control shRNA adenovirus and TRIM47 shRNA adenovirus (*n* = 8). **h** Tumor volume was measured after different treatments in nude mice (*n* = 8, non-parametric Mann-Whitney test, *P* < 0.05). **i** Tumor weight was measured in nude mice after different treatments (n = 8, non-parametric Mann-Whitney test, *P* < 0.05). **j** Representative images of Ki67 IHC staining of the three groups mice xenografts (*n* = 8, non-parametric Mann-Whitney test, *P* < 0.05). **k** Proliferation related proteins c-Myc and Cyclin D1 were detected in mice xenografts by western blot (*n* = 3)

We found that knockdown of TRIM47 notably suppressed cell proliferation both in HCT116 and HT29 cells (Fig. 3a and b), while overexpression of TRIM47 increased cell proliferation ability of SW480 cells (Fig. 3c). Furthermore, colony formation assay showed that knockdown of TRIM47 could sharply inhibit the colony formation in HCT116 and HT29 cells, reflected by fewer and smaller colonies in the TRIM47 siRNA-treated group (Fig. 3d). Consistently, colony forming capacity in TRIM47-overexpressing group was dramatically increased compared to cells treated with empty vector in SW480 (Fig. 3e). The protein levels of proliferation related proteins, C-Myc and Cyclin D1, were evaluated by western blot. As shown in Fig. 3f, the levels of C-Myc and Cyclin D1 were reduced in TRIM47 knockdown cells and increased in TRIM47-overexpressing group. Next, we found that downregulation of TRIM47 remarkably reduced CRC tumor growth (Fig. 3g and h) and tumor weight (Fig. 3i) in xenograft mouse tumor model. In support of the pro-tumor role of TRIM47, the Ki67 staining showed that knockdown of TRIM47 decreased tumor cell proliferation in vivo (Fig. 3j). The protein levels of proliferation related proteins, C-Myc and Cyclin D1, were reduced in TRIM47 knockdown group (Fig. 3k). These results suggested that inhibition of TRIM47 expression impaired colorectal cancer cell proliferation in vitro and in vivo.

**Fig. 4:**
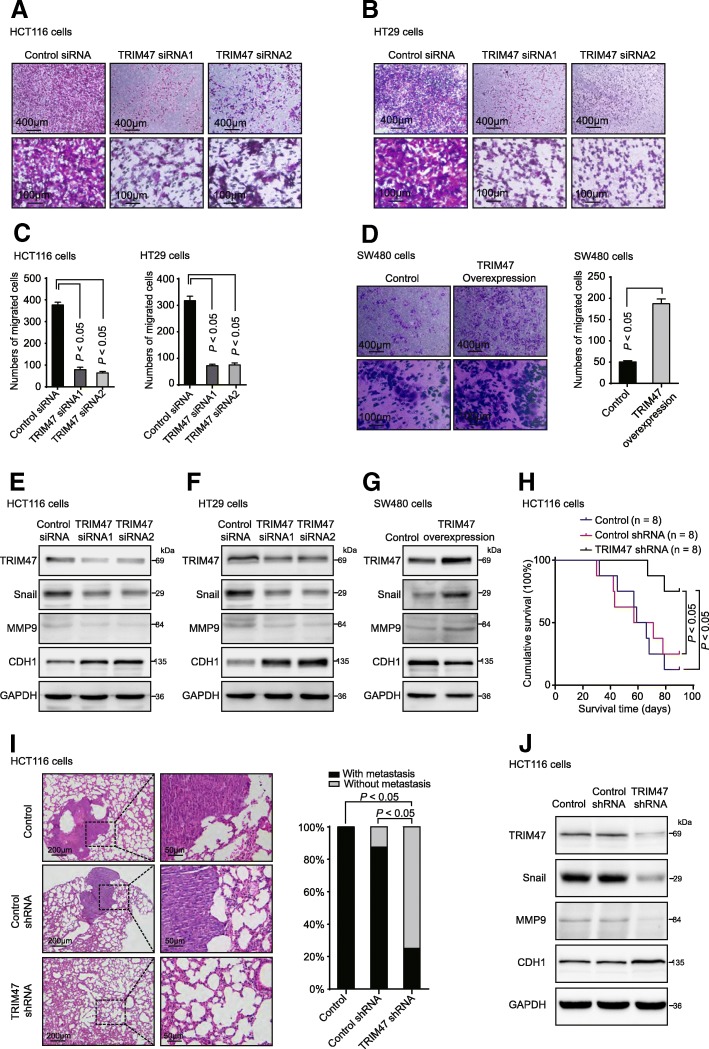
TRIM47 promotes metastasis of colorectal cancer cells in vitro and in vivo. **a**-**c** Transwell invasion assay was performed in HCT116 and HT29 cells transfected with control siRNA and TRIM47 siRNAs (*n* = 3, non-parametric Mann-Whitney test, *P* < 0.05). **d** Transwell invasion assay was performed in SW480 cells transfected with vector plasmids and TRIM47 overexpression plasmids (*n* = 3, non-parametric Mann-Whitney test, *P* < 0.05). **e**-**g** Metastasis related proteins Snail, MMP9 and CDH1 were detected in HCT116, HT29 and SW480 cells by western blot (*n* = 3). **h** Survival analysis in nude mice bearing colorectal cancer cells injected with PBS, control shRNA adenovirus and TRIM47 shRNA adenovirus, respectively (n = 8, Log-rank test, *P* < 0.05). **i** Representative HE staining and summarized data on tumor lung foci in nude mice at 14 weeks after treatment with PBS, control shRNA adenovirus and TRIM47 shRNA adenovirus (*n* = 8, non-parametric Mann-Whitney test, *P* < 0.05). **j** Metastasis related proteins Snail, MMP9 and CDH1 were detected in mice tumor tissue by western blot (*n* = 3)

Furthermore, we examined the effects of TRIM47 on colorectal cancer cell invasion and metastasis function. In the invasion assay, we revealed that knockdown of TRIM47 significantly reduced the invasion ability in HCT116 and HT29 cells (Fig. 4a-c). In the gain-of-function assay, overexpression of TRIM47 increased the invasion ability of SW480 cells (Fig. 4d). The protein expression levels of metastasis-related proteins, Snail, MMP9 and CDH1, were evaluated by western blot. As shown in Fig. 3f, the levels of Snail and MMP9 were sharply reduced in TRIM47 knockdown groups and increased in TRIM47-overexpressing group, CDH1 level was on the contrary (Fig. 4e-g). To further validate the effect of TRIM47 on colorectal cancer cell metastasis, we conducted the CRC metastatic mouse model. The result showed that the mice inoculated with TRIM47 shRNA adenovirus had a longer overall survival time than that treated with control shRNA or Phosphate Buffered Solutions (PBS) (Fig. 4h). The metastatic foci in the lungs of nude mice at 14 weeks after injection of TRIM47 shRNA adenovirus were fewer compared with the control groups (Fig. 4i). The protein levels of metastasis related proteins, Snail and MMP9 were reduced in TRIM47 knockdown group and the CDH1 expression was on the contrary (Fig. 4j). These data strongly suggested that TRIM47 may promote colorectal cancer progression by regulating colorectal cancer cell proliferation and metastasis.

### TRIM47 upregulates the expression of CCL15 and CCR1 via interacting with SMAD4 and enhancing ubiquitylation and degradation of SMAD4

**Fig. 5:**
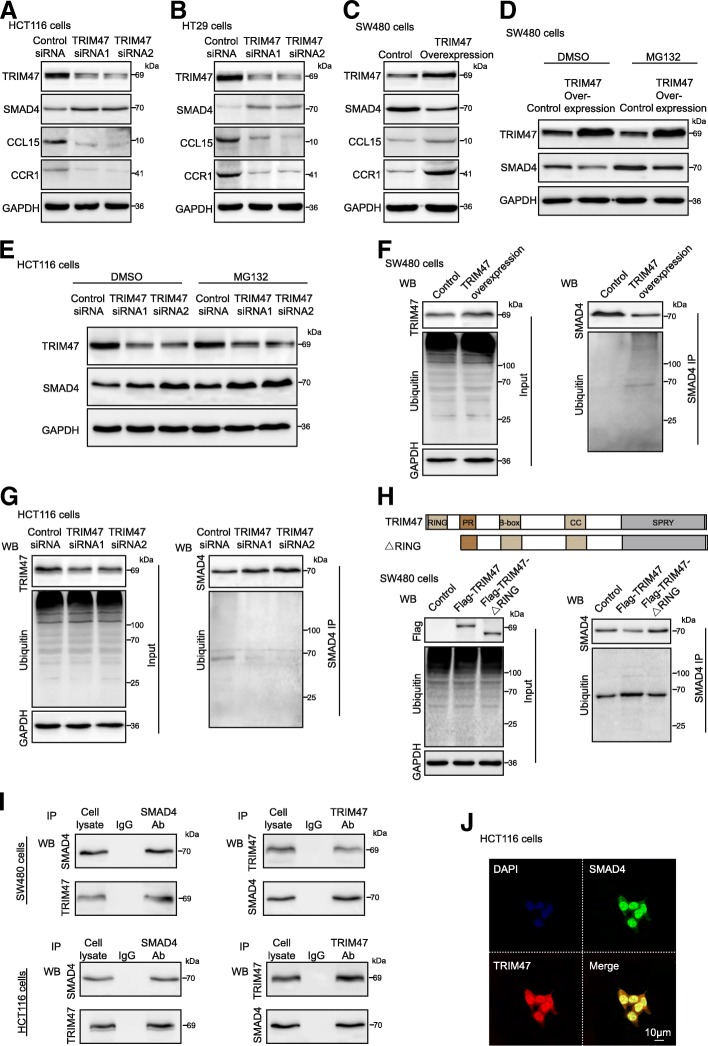
TRIM47 upregulates the expression of CCL15 and CCR1 via interacting with SMAD4 and enhancing ubiquitylation and degradation of SMAD4. **a** and **b** Western blot was performed in HCT116 and HT29 cells transfected with control siRNA and TRIM47 siRNAs (*n* = 3). **c** Western blot was performed in SW480 cells transfected with vector plasmids and TRIM47 overexpression plasmids (n = 3). **d** Western blot was performed in SW480 cells transfected with TRIM47 overexpression plasmids and treated with DMSO or MG132 (*n* = 3). **e** Western blot was performed in HCT116 cells transfected with TRIM47 siRNAs and treated with DMSO or MG132 (*n* = 3). **f** The amount of ubiquitin that co-immunoprecipitated with SMAD4 in SW480 cells transfected with TRIM47 overexpression plasmids. Western blot data of TRIM47, ubiquitin and GAPDH from 20% input (left). Anti-ubiquitin and anti-SMAD4 antibody were used for western blot to determine the ubiquitination level of SMAD4 (right) (*n* = 3). **g** The amount of ubiquitin that co-immunoprecipitated with SMAD4 in HCT116 cells transfected with TRIM47 siRNAs (*n* = 3). **h** The amount of ubiquitin that co-immunoprecipitated with SMAD4 in SW480 cells transfected with TRIM47 overexpression plasmids and the △RING domain plasmids (*n* = 3). RING, RING-finger domain; PR, proline-rich region; B-box, B-box-type zinc finger; CC, coiled coil region; SPRY, C-terminal SplA/ryanodine receptor domain; Flag-, Flag-tagged; △RING, deletion of the RING domain. **i** Co-immunoprecipitation detected the interaction of TRIM47 and SMAD4 in SW480 and HCT116 cells. The specific immunoprecipitation of TRIM47 and SMAD4 was confirmed by western blot (*n* = 3). **j** Immunofluorescence revealed that TRIM47 is co-localized with SMAD4 (*n* = 3)

To understand the molecular mechanism by which TRIM47 promotes cell proliferation and invasion of colorectal cancer, we examined the protein expression of SMAD4. The SMAD4 gene is located in human chromosome 18q21, a region with frequent genetic loss of heterozygosity in CRC [18, 19]. Loss of SMAD4 is seen in 30–40% of CRCs [20] and is associated with distant metastases, a poor prognosis and drug resistance [21, 22]. We examined the SMAD4 protein expression by immunohistochemical staining assay in 180 paraffin-embeddded colorectal cancer and adjacent normal tissues (Cohort 2). SMAD4 protein expression was lower in colorectal cancer tissues than the adjacent tissues (*P* < 0.05, Additional file 1: Figure S1A and B). Next, univariate and multivariate regression analysis of cohort 2 were performed to identify the risk factors associated with patient overall survival. The results revealed that SMAD4 expression was an independent predictor of colorectal cancer aggressiveness with significant hazard ratios for predicting clinical outcome (Additional file 1: Figure S1C and D). To date, most research in regard to the role of SMAD4 in CRC has focused exclusively on its role as part of the TGFβ signal pathway. However, loss of SMAD4 was also reported to lead to up-regulation of CCL15 expression and cause the poor outcome in human CRC through the CCL15-CCR1signaling [23, 24]. We first determined the expression levels of SMAD4, CCL15 and CCR1 in human CRC cells HCT116 and HT29. As shown in Fig. 5a and b, the expression of SMAD4 was increased in HCT116 and HT29 cells, whereas the expression levels of CCL15 and CCR1 were decreased with TRIM47 knockdown. In addition, overexpression of TRIM47 significantly down-regulated SMAD4 level and up-regulated CCL15 and CCR1 in SW480 cells (Fig. 5c). Next, we explored the molecular mechanism by which TRIM47 regulated SMAD4 protein expression level. Given the fact that TRIM47 plays the E3 ubiquitin ligase role [17] and SMAD4 could be ubiquitinated and degraded by the ubiquitin-specific protease(USP) 4 [25], we hypothesized that TRIM47 may downregulate SMAD4 expression via promoting SMAD4 protein ubiquitination and degradation. To certify the hypothesis, we used MG132 (a proteasome inhibitor) to treat human CRC cells. We found that MG132 treatment distinctly rescued TRIM47-induced downregulation of SMAD4 in SW480 cells compared with DMSO treatment (Fig. 5d). These data were further confirmed in loss-of-function assays in HCT116 cells (Fig. 5e). As shown in Fig. 5f, the amount of ubiquitin that co-immunoprecipitated with SMAD4 was obviously increased in SW480 cells with TRIM47 overexpression. Consistently, knockdown of TRIM47 impaired SMAD4–ubiquitin association in HCT116 cells (Fig. 5g). To further illustrate the E3 ligase activity of TRIM47, we construct the expression plasmids encoding TRIM47 with deletion of the RING domain. As shown in Fig. 5h, overexpression of full-length TRIM47 induced ubiquitination of SMAD4, whereas deletion of the RING domain of TRIM47 (△RING) abrogated this capability, in SW480 cell. In addition, we observed that TRIM47 and SMAD4 interacted with each other in SW480 and HCT116 cells (Fig. 5i). Subsequently, confocal microscopy revealed that SMAD4 and TRIM47 colocalized in SW480 cells (Fig. 5j). Together, these data indicate that TRIM47 serves as a E3 ubiquitin ligase for SMAD4–ubiquitin association.

**Fig. 6:**
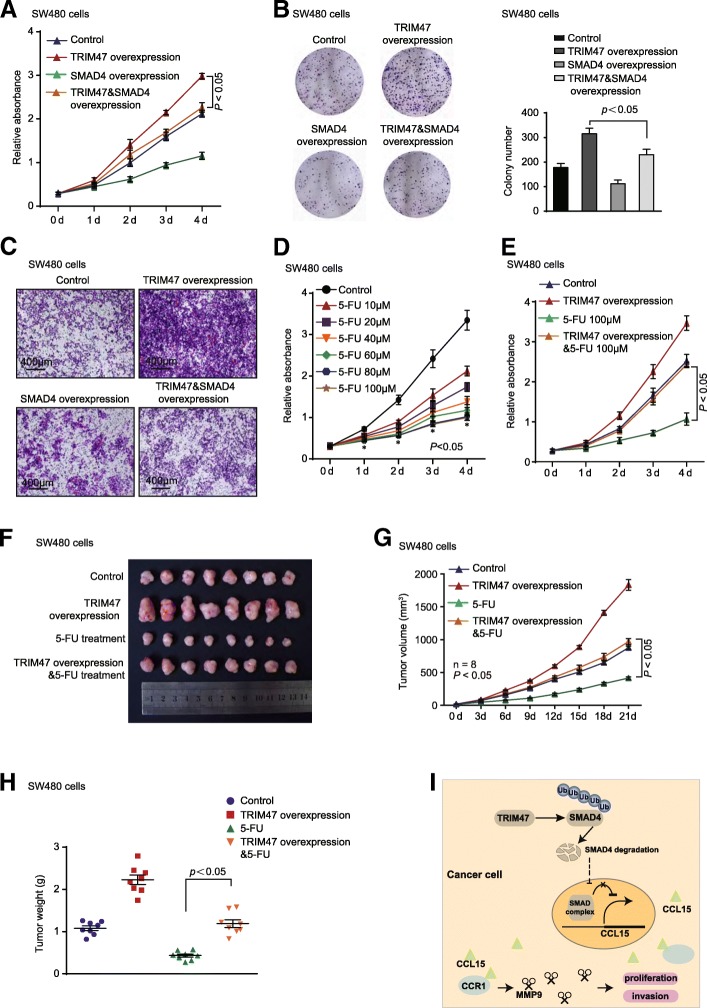
TRIM47 overexpression promotes 5′-fluorouracil resistance of colorectal cancer cells in vitro and in vivo. **a** and **b** Cell proliferation assay was performed in SW480 cells transfected with vector plasmids, TRIM47 overexpression plasmids and SMAD4 overexpression plasmids (n = 3, non-parametric Mann-Whitney test, *P* < 0.05). **c** Cell transwell invasion assay was performed in SW480 cells transfected with vector plasmids, TRIM47 overexpression plasmids and SMAD4 overexpression plasmids (*n* = 3, non-parametric Mann-Whitney test, *P* < 0.05). **d** Cell proliferation assay was performed in SW480 cells treated with increasing concentrations of 5-FU. (*n* = 3, non-parametric Mann-Whitney test, *P* < 0.05). **e** Cell proliferation assay was performed in SW480 cells transfected with vector plasmids, TRIM47 overexpression plasmids and 5-FU (*n* = 3, non-parametric Mann-Whitney test, *P* < 0.05). **f** Representative images of tumors in nude mice bearing colorectal cancer cells treated with control adenovirus, TRIM47 overexpression adenovirus, 5-FU, TRIM47 overexpression adenovirus & 5-FU (*n* = 8). **g** Tumor volume was measured after different treatments in nude mice (*n* = 8, non-parametric Mann-Whitney test, *P* < 0.05). **h** Tumor weight was measured in nude mice after different treatments (*n* = 8, non-parametric Mann-Whitney test, *P* < 0.05). **i** A schematic model of TRIM47 function in colorectal carcinogenesis. TRIM47 interacts with SMAD4 and promotes the ubiquitination and degradation of SMAD4, impairing the suppression of CCL15 and CCR1, ultimately promoting colorectal cancer cells proliferation and metastasis

Next, we hypothesized that TRIM47 may act as an oncogene by ubiquitylating and degrading SMAD4, and then eventually enhancing CCL15 expression in CRC. To verify this hypothesis, SMAD4 overexpression plasmid was transfected into colorectal cancer cells and we examined the effects on cancer cell biological functions. The results showed that overexpression of SMAD4 significantly reduced colorectal cancer cell proliferation and invasion induced by TRIM47 in SW480 cells (Fig. 6a-c).

### TRIM47 overexpression promotes 5′-fluorouracil resistance of colorectal cancer cells in vitro and in vivo

Since increased research showed that E3 ubiquitin ligase was correlated with drug resistance [26, 27], we hypothesized that TRIM47 overexpression might affect the chemotherapeutics response of colorectal cancer cells in vitro and in vivo. As shown in Fig. 6d, 5′-fluorouracil (5-FU) could inhibit cell proliferation in a dose-dependent manner in SW480 cells. Interestingly, when cells were treated with both 5-FU and TRIM47 overexpression plasmid, the suppression of proliferation function was remarkably impaired compared with that treated with only 5-FU (Fig. 6e). In the CRC xenograft mouse models, SW480 cells were inoculated into nude mice, followed by treated with control adenovirus, TRIM47 overexpression adenovirus, 5-FU, TRIM47 overexpression adenovirus & 5-FU. We found that tumor growth was significantly decreased by 5-FU treatment, and these decreases were blocked by TRIM47 overexpression adenovirus treatment in vivo (Fig. 6f-h). Thus, TRIM47 played a role in CRC chemoresistance in response to 5-FU therapy.

## Discussion

Accumulating studies have shown that TRIM proteins positively and negatively regulate carcinogenesis [28, 29]. Recent pathological analysis has shown that changes in the expression of some TRIM proteins are strongly associated with the malignancy of cancers and prognosis [30]. The present study provides experimental evidence that TRIM47 is overexpressed significantly in colorectal cancer. Obviously shortened overall survival is seen in patients with high TRIM47 expression compared with those with low TRIM47 expression. In cultured colorectal cancer cells and xenograft nude mouse models, downregulation of TRIM47 markedly suppresses colorectal cancer cell proliferation and metastasis. The data consistently demonstrated that high TRIM47 expression may function as an oncogene in human colorectal carcinogenesis.

TRIM proteins usually participate in the progression of cancers by ubiquitylating and degrading target proteins [31]. However, the underlying molecular mechanisms of TRIM47 in colorectal cancer cell growth and metastasis have never been investigated before. SMAD4 is one of the genes located in human chromosome 18q21 where frequent loss of heterozygosity is observed in CRC progression as a poor prognostic marker [32, 33]. The Cancer Genome Atlas (TCGA) research network has identified mutations in SMAD4 to be among the most frequently mutated genes in colon cancers [34]. To date, most research report that SMAD4 serves as a common downstream node of transforming growth factor β (TGFβ) and bone morphogenetic protein (BMP) pathways [35]. However, loss of SMAD4 is also reported to lead to up-regulation of CCL15 expression and cause the poor outcome in human CRC through the CCL15-CCR1 signaling [23, 24]. Accumulating evidence has suggested that CCL15, a member of the CC chemokine family and expressed only in the gut and the liver, may have a crucial role in the progression of tumor cells via the CC chemokine receptor [36]. CCR1, the main and specific receptor for CCL15, is a G protein-coupledreceptor that is expressed by a variety of cells such as monocytes, lymphocytes, neutrophils, eosinophils [37] and hepatocellular carcinoma cells [38]. In human CRC cells, loss of SMAD4 leads to up-regulation of CCL15 expression. Human liver metastases that express CCL15 contain higher numbers CCR1^+^ cells; patients with these metastases have shorter times of disease-free survival [23]. In addition, CCR1 knockdown significantly limits the activity and expression of matrix metalloproteinase-2 (MMP-2) and MMP-9 [36], which predicts poor survival in colorectal cancer [39]. In this study, we have illustrated the mechanisms by which TRIM47 plays the role in CRC cells proliferation and metastasis. TRIM47 physically binds to SMAD4 and enhances the ubiquitination and degradation of SMAD4. This point is supported by three lines of experimental evidence. First, knockdown of TRIM47 increased the expression of SMAD4 in colorectal cancer cells and the data were verified in gain function assay as well. Second, MG132 treatment, the inhibitor of proteasome, disrupted TRIM47-induced SMAD4 downregulation in colorectal cancer cells. Last, the association between SMAD4 and ubiquitin was reduced by TRIM47 downregulation in the coimmunoprecipitation (co-IP) data, and TRIM47 upregulation leaded to a significant increase of the ubiquitin that co-IP with SMAD4. Accumulating loss of SMAD4 leads to up-regulation of CCL15 and CCR1 expression, followed by higher activity and expression of MMP-9, ultimately causing colorectal cancer malignance (Fig. 6i).

In addition to its biological importance, our work may be relevant in clinical management of patients with colorectal cancer. Survival analyses illustrate that TRIM47 overexpression can predict poor clinical outcome in patients with colorectal cancer from Renji cohort, which indicate that the measurement of TRIM47 expression may be an effective approach to predict patient prognosis, and TRIM47 may be a promising diagnostic biomarker and therapeutic target in patients with colorectal cancer. We show evidence in this study that the CRC cells growth was significantly decreased by 5-FU treatment, and these decreases were blocked by TRIM47 overexpression treatment in vitro and in vivo*.* TRIM47 played a role in CRC chemoresistance in response to 5-FU therapy. Thus, it is important to detect TRIM47 expression and its associated pathway to improve the sensibility to chemotherapy.

## Conclusions

In summary, our study has shown the biological and clinical significance of TRIM47 in colorectal cancer. TRIM47 exerts an inhibitory effect on SMAD4 by ubiquitylating and degrading SMAD4, thereby promoting tumor growth and progression. TRIM47 could be regarded as a biomarker to guide early diagnosis and therapy in colorectal cancer patients, and pharmaceutical intervention to TRIM47 expression may provide a promising strategy to improve the outcome of CRC.

## Additional file

Additional file 1: **Figure S1.** SMAD4 is loss in colorectal cancer and correlated with poor prognosis. (A) Representative images of SMAD4 protein expression in colorectal cancer tissues and paired adjacent tissues using IHC in Renji dataset (Cohort 2, *n* = 180). (B) Statistical analysis of SMAD4 protein expression in colorectal cancer tissues and paired adjacent tissues using IHC staining in Renji dataset (Cohort 2, *n* = 180, nonparametric Mann–Whitney test, *P* < 0.05). (C) Univariate regression analysis in Cohort 2 (All the bars correspond to 95% confidence intervals). (D) Multivariable regression analysis in Cohort 2 (All the bars correspond to 95% confidence intervals). (PDF 356 kb)

Additional file 2: Clinial information of 100 cases CRC patients (fresh tissues). (XLSX 16 kb)

Additional file 3: Clinial information of Female8Male cases CRC patients (paraffin tissues). (XLSX 21 kb)

## Abbreviations

5-FU: 5′-fluorouracil
BMP: Bone morphogenetic protein
CCL15: C-C motif chemokine ligand 15
CCR1: C-C motif chemokine receptor 1
CDH1: Cadherin 1
Co-IP: Coimmunoprecipitation
CRC: Colorectal cancer
MMP9: Matrix metalloproteinase 9
TCGA: The Cancer Genome Atlas
TGFβ: Transforming growth factor β
TRIM: Tripartite motif
UPS: Ubiquitin–proteasome system
