# Supplementary figures and images for: TRIM47 is up-regulated in colorectal cancer, promoting ubiquitination and degradation of SMAD4
Source: J Exp Clin Cancer Res. 2019 Apr 12;38:159. doi: 10.1186/s13046-019-1143-x (PMC6461818; doi:10.1186/s13046-019-1143-x)

Supplementary Figure 1

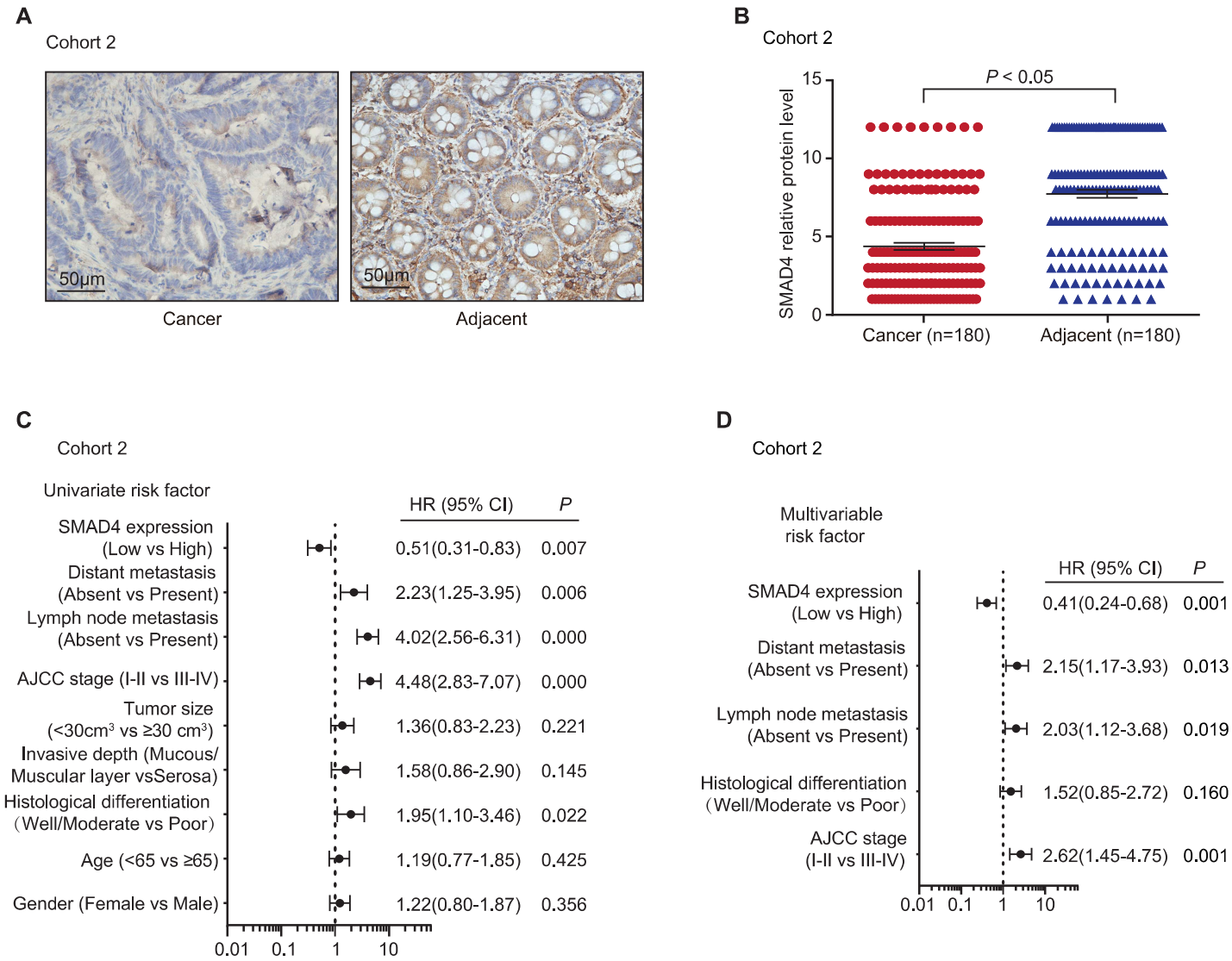

Supplement: Supplementary file 1 — Figure S1. SMAD4 is loss in colorectal cancer and correlated with poor prognosis. (A) Representative images of SMAD4 protein expression in colorectal cancer tissues and paired adjacent tissues using IHC in Renji dataset (Cohort 2, n = 180). (B) Statistical analysis of SMAD4 protein expression in colorectal cancer tissues and paired adjacent tissues using IHC staining in Renji dataset (Cohort 2, n = 180, nonparametric Mann–Whitney test, P < 0.05). (C) Univariate regression analysis in Cohort 2 (All the bars correspond to 95% confidence intervals). (D) Multivariable regression analysis in Cohort 2 (All the bars correspond to 95% confidence intervals). (PDF 356 kb) [file 13046_2019_1143_MOESM1_ESM.pdf]
